# Mapping Sonification for Perception and Action in Motor Skill Learning

**DOI:** 10.3389/fnins.2017.00463

**Published:** 2017-08-21

**Authors:** John F. Dyer, Paul Stapleton, Matthew Rodger

**Affiliations:** ^1^School of Psychology, Queen's University Belfast Antrim, United Kingdom; ^2^Sonic Arts Research Centre, School of Arts, English and Languages, Queen's University Belfast Antrim, United Kingdom

**Keywords:** sonification, motor skill learning, augmented feedback, concurrent feedback, guidance effect, perception and action

## Introduction, goal, and scope

Real-time sonification of human movement (conversion of motion signals into sound) can be used as augmented feedback for motor skill learning. With sonification, motor skills can, in some instances, be learned more quickly and successfully (Sigrist et al., 2013a). The goal of such sonification systems is a permanent (or at least, lasting) improvement in performance at a physical task or skill, which persists in the absence of augmented feedback. Many experimental investigations of feedback, however, show that when performance is tested without feedback, a decline occurs (Park et al., 2000; Maslovat et al., 2009). This finding has become known as the “guidance effect” (Buchanan and Wang, 2012). It has been suggested that this effect is a consequence of learner overreliance on the “guidance” provided by augmented feedback, at the expense of task-intrinsic sensory feedback. For effective learning, this is clearly not desirable.

In this paper, we advocate a perception-action approach to sonification when used as feedback for skill learning, which may lead researchers and trainers to design more effective prototypes. We highlight three main issues: 1. The learner's task should be conceived as perception-action based and sonification designed accordingly, 2. Sonification should provide Ecological information for perception rather than propositional knowledge-of-performance, and 3. Ecologically meaningful sound morphologies should be harnessed effectively.

## Performance

Successful coordination requires the pickup and use of event-structured information through perception and action (Gibson, 1972). Perceptual information available to a moving agent can be said to specify the state of the agent-environment system (i.e., the task); this enables the skilled agent to control movement and perceive its outcome “directly” (Warren, 2006; for a formal explanation of the relation between Ecological information and task dynamics, see Turvey et al., 1981). Through repeated interactions with a task, novices can become selectively sensitive to informational variables which best serve task goals, and become more adept at bringing these variables into use for coordination—a process described by Eleanor Gibson as “education of attention” (Gibson, 1969; Jacobs and Michaels, 2007). Motor skill learning is therefore characterized by the “tuning in” of perception and action—a progression toward the active pickup of better-specifying and more useful informational variables (Huys et al., 2009; Gray, 2010; Wilson et al., 2010a). Stoffregen and Bardy (2001) propose that action is controlled via the pickup of multisensory informational variables, which better specify the state of the perception-action system than can unimodal variables. Sonification can therefore provide higher-order information—available via interaction and specific to that interaction—which enables better perception and control of movement. With sonification, novices can practice with an enhanced, more responsive perceptual-motor workspace (defined as the emergent resources and constraints of organism and environment in the context of a task, which are perceptually available through dynamic interaction: see Newell et al., 1991), which employs sound as a helpful constraint on action.

A model of the informational variables available and useful for the learner in a task can be guided by existing literature, or refined by pilot testing. However, the most useful informational variable(s) for the perceiving learner may not necessarily correspond to the motor variable being tracked by the researcher as a measure of performance. In other words, measurement and experience are not isomorphic. As an example, consider research on sonified reaching and target tracking (Oscari et al., 2012; Schmitz and Bock, 2014; Boyer et al., 2016). The task as instantiated here is to track or reach for a target, while using whatever information is provided by the system to guide one's effector/pointer. The variable of interest for measurement in this task (and others) is the absolute positional difference between hand/pointer position and target position (error). This variable is frequently sonified, by mapping error to a sonic variable such as pitch, amplitude or inter-aural panning, with mixed results (Konttinen et al., 2004; Rosati et al., 2012; Sigrist et al., 2013b). It is not certain that instantaneous positional error is a relevant variable for a moving individual in an everyday context. Everyday pointing for example, is primarily a visuomotor task with a criterion for success often defined in social terms (Kennedy, 1985). It makes sense from the detached perspective of an experimenter to measure positional error as an objective performance index, but perhaps another, possibly higher-order variable might be more useful for the learner as a perceiver (Runeson, 1977). The choice of what to sonify may have additional implications for the guidance effect, as the next section will detail.

## Learning and the guidance effect

An analysis of the task can enable identification of the perception-action resources used by a skilled performer in an everyday context, including important informational variables and control parameters (Wilson and Golonka, 2013; Bruineberg and Rietveld, 2014). Sonification systems can then be designed to *highlight* these same useful variables/parameters, rather than to create new parameters—control of which might be independent of how the task would be performed without feedback. The value of highlighting task-intrinsic information lies in the possibility to avoid the guidance effect of augmented feedback. As an example, Ronsse et al. (2011) provided direct sonification of changes in hand-movement direction with a set of two tones. Participants were required to learn a 90° out-of-phase bimanual wrist coordination task in which a two-tone isochronous galloping rhythm was produced by perfect performance. When sonification was withdrawn, participant performance remained stable. In contrast, a second group of participants who had practiced with movement-coupled graphical feedback showed a decline in performance following withdrawal. In this task, sonification preserved the spatio-temporal structure of relevant task-intrinsic events in the perceptual-motor workspace, therefore the information required to control movement was perceivable with or without feedback. Sonification had acted as a guide for its pickup. Conversely, graphical feedback provided information for the direct control of bimanual phase-relationship; its removal meant that coordination was no longer possible as the required information was absent (see Wilson et al., 2010b). If learning is seen as education of attention (Jacobs and Michaels, 2007), the need to sonify task-intrinsic events to avoid the guidance effect is clear (for similar findings, see Dyer et al., 2017).

## Knowledge and information

Current understanding of augmented feedback and its role in performance enhancement has its foundations in classic studies on knowledge-of-results and knowledge-of-performance (KR/KP) feedback (Adams, 1971). Today, sonification in Psychology is still widely discussed using these terms (Konttinen et al., 2004; Dyer et al., 2015; Sors et al., 2015; Fujii et al., 2016). However, this continuity belies a subtle shift in what these terms have come to mean over time as technology has improved to the point where real-time sonification as KP is possible. In the late twentieth Century, both KP and KR meant explicit, propositional knowledge—typically verbal (or verbalisable) knowledge about movement outcome (KR) or performance (KP). Older reviews of KP/KR research (Adams, 1971; Salmoni et al., 1984) show that motor skill learning was explicitly conceptualized as a knowledge and memory-based, problem-solving task, soluble by the application of explicit knowledge and rules (typically, coach-provided guidance, or scores/graphs of performance and error). The goal was to improve performance by delivering instructions, which could be applied to programming of motor output “intellectually,” i.e., independently of perception and action. Thomas and Thomas (1994) have argued that the traditional knowledge-based approach to motor skill learning underplays the role of selective sensitivity to perceptual information in skilled performance, catering mostly to the earliest “cognitive stage” of learning (Fitts and Posner, 1967).

Today, augmented feedback (including sonification) is often delivered concurrently with movement (Sigrist et al., 2013a), and could be considered something more like augmented information for online, perceptual control of action. However, the older style of thinking about feedback as explicit knowledge is evident in many modern implementations. This thinking manifests in the design of mappings intended to transmit a signal to the learner, which can be said to *contain* knowledge—in a description of current performance relative to an ideal (often a sonified error score). This signal must be parsed by the learner with the application of a remembered mapping rule, and the decoded knowledge applied to update ongoing movement. This bears directly on sonified feedback given the requirement for learner interpretation associated with such rule-based mappings. Time required to interpret the knowledge contained in an auditory error signal puts an intellectual barrier between perception and action, which may not be conducive to fluid, skilful performance. The perception-action approach contends that “knowledge” related to skill is primarily enacted via perception-action engagement with the dynamics of a task—rather than through the rote application of schemas and rules (for this argument, see Ingold, 2000, 2001; van Dijk et al., 2015). Effenberg and colleagues (Vinken et al., 2013; Effenberg et al., 2016) argue similarly that a “direct” approach to mapping in which sound quality perceptually correlates with the dynamics of ongoing movement is most appropriate for motor skill learning. Learners can learn to use sonic information to perceive movement directly, with no need for cognitive elaboration, as the control of movement is directly related to the sensory consequences of movement (for a related example, see Stienstra et al., 2011). This approach preserves the immediacy of Ecological perception, and “knowledge” emerges from two-way interaction rather than being translated from an incoming coded signal.

## Ecologically-meaningful sound

A theme in this article has been that sonification researchers interested in motor skill learning should understand that learners are primarily perceivers, with pre-existing skills. Perception of something meaningful, or action-relevant in a sonic experience can be conceptualized as an active listening skill which is related to the sociocultural context of its development (Steenson and Rodger, 2015). It follows then that listeners might already know how to listen and interact with certain sound morphologies, in certain contexts/tasks. The use of sounds which cater to existing bodily skills, such as physical modeling of metallic scraping in a writing task (Danna et al., 2015) constrain the learner's relation to the task and guide perception and action more effectively than might be possible with more basic pitch mapping (see also Roddy and Furlong, 2014). However, Dubus and Bresin (2013) show that pitch mapping (of a pure tone or the center frequency of filtered noise) remains a common strategy in sonification generally, but also in sonification of motor tasks. Most individuals have little experience using a pure tone for movement coordination, therefore such a mapping may be challenging and require extensive training before it can be used. The use of already-familiar sound morphologies (e.g., melodies, rhythms, sounds of real-life noisy interactions) may produce more “intuitive” feedback systems. What we advocate here is not a distinction which is sometimes made, between “ecological” sounds of the natural world on the one hand and “artificial,” synthetic sounds on the other. “Meaningfulness,” in an Ecological sense, is defined relative to a perceiver's experience using the information which a sound source provides.

## Conclusion

In this paper, we have argued for a perception-action approach to motor skill learning as the basis for understanding the utility of sonification as augmented feedback. If information supports performance, then sonification should highlight task-intrinsic information to counter the guidance effect. A clearer definition of what “information” is can help guide the design of sonified feedback whereby knowledge is a product of interaction rather than transmission (e.g., see Wilson and Golonka, 2013). Lastly, learners have abundant socioculturally-situated listening experience already; it is therefore undershooting the potential of sonification as feedback to rely only on unfamiliar or esoteric sound morphologies like pure tones. To speculate, the common root of these three issues for design may be related to the existence of different frames of reference for the experimenter/designer and the learner. It is more helpful to see the learner as a situated agent with a repertoire of existing perception-action skills than an engine that must apply propositional knowledge to enact a desired change in state.

## Author contributions

JD conceived the topic in discussion with PS and MR and also drafted the manuscript. All three authors were involved in redrafting and editing of the manuscript.

### Conflict of interest statement

The authors declare that the research was conducted in the absence of any commercial or financial relationships that could be construed as a potential conflict of interest.
